# Effect of Vitamin C on mortality of critically ill patients with severe pneumonia in intensive care unit: a preliminary study

**DOI:** 10.1186/s12879-021-06288-0

**Published:** 2021-06-29

**Authors:** Ata Mahmoodpoor, Kamran Shadvar, Sarvin Sanaie, Mir Reza Hadipoor, Mohammad Ata Pourmoghaddam, Seied Hadi Saghaleini

**Affiliations:** 1 Fellowship of critical care medicine, Faculty of Medicine, Tabriz University of Medical Sciences, Tabriz, Iran; 2 Neurosciences Research Center, Aging Research Institute, Tabriz University of Medical Sciences, Tabriz, Iran; 3 Student Research Committee, Tabriz University of Medical Sciences, Tabriz, Iran; 4 Yeditepe Dental Student Association Research Committee Member, Faculty of Dentistry, Yeditepe University, Istanbul, Turkey

**Keywords:** Vitamin C, Pneumonia, Mortality, Critically ill

## Abstract

**Background:**

Critically ill patients frequently suffer from vitamin C deficiency. Previous studies showed that high doses of vitamin C administration had conflicting results on clinical outcomes in patients with severe sepsis, burns, and trauma. Because of the high incidence and morbidity/mortality with severe pneumonia, we aimed to investigate the effect of administration of high dose vitamin C in critically ill patients with severe pneumonia.

**Methods:**

Eighty critically ill patients with pneumonia were enrolled in this randomized double-blinded clinical trial. Patients with a CURB-65 score > 3, one major criterion, or ≥ 3 minor criteria were considered as severe pneumonia. Patients were randomly assigned to intervention or placebo groups receiving standard treatment plus 60 mg/kg/day vitamin C as a continuous infusion or normal saline in the same volume correspondingly for 96 h. Serum levels of vitamin C were noted at baseline and 48 h after vitamin C administration. Duration of mechanical ventilation, ICU length of stay, PaO_2_/FiO_2_, and mortality rate were noted for all patients till the 28th day. Any complications related to the vitamin C administration were recorded.

**Results:**

Duration of mechanical ventilation and vasopressor use were significantly lower in the intervention group (*p*: < 0.001 and 0.003, respectively). Baseline levels of vitamin C in both groups did not have a significant difference but its levels increased in the intervention group and decreased in the control group during the study period. Mortality rate insignificantly decreased in the intervention group (*p* = 0.17). Three patients showed hypotension and tachycardia during the administration of vitamin C which was self-limited with decreasing the dose of vitamin C.

Our results showed that the intravenous administration of a relatively high dose of vitamin C to critically ill patients with severe pneumonia was safe and could decrease the inflammation, duration of mechanical ventilation, and vasopressor use without any significant effect on mortality.

Trial registration: IRCT registration number: IRCT20190312043030N1, Registration date: 2019-08-26, Seied Hadi Saghaleini.

## Background

Plasma levels of vitamin C are usually decreased in critically ill patients with sepsis, burn, major surgeries, trauma, and any disease accompanied by immune dysfunction and inflammation [1]. This reduction in Vitamin C level is contributed to lower intake and higher metabolism in critically ill patients [2]. Based on the modulation of oxidative stress, endothelial protection, and involvement in organ functionality and energy metabolism, vitamin C represents an interesting therapeutic approach in critically ill patients as a safe and low-cost nutrient. Recently, there are many published trials about the positive effect of combination therapy of high dose vitamin C, thiamin, and fludrocortisone in septic patients. They showed that this combination which targeted multiple components of host response could synergistically restore the immune dysfunction; however, future studies showed different results regarding morbidity and mortality [3, 4]. Based on the high incidence of pneumonia in critically ill patients and its effect on mortality, it seems that the effects of vitamin C on respiratory infections are also important at the level of fundamental concepts. Corkovic et al. showed that the serum level of vitamin C significantly decreased in patients with acute pneumonia and patients with exacerbation of COPD. They also showed a negative correlation between the level of vitamin C and laboratory markers of inflammation [5]. Results of a systematic review in 2004 showed that vitamin C substantially reduced the incidence or severity of respiratory infections but there were many questions about the heterogeneity of trials, route and dose of vitamin C administration, and sample size of the studies [6]. Results of a recently published Cochrane systematic review showed skepticism about the effect of vitamin C supplementation on the prevention and treatment of pneumonia. They also emphasized conducting better-quality studies for assessment of the role of vitamin C supplementation in the prevention and treatment of pneumonia [7]. Considering the dosing and route of administration is a very important issue in vitamin C therapy of critically ill patients. It is unclear whether the dosing strategy should attempt to achieve normal or supraphysiologic plasma vitamin C levels. On the other hand, the intravenous dose is necessary and enteral uptake due to gut dysfunction is unpredicted [8]. Recent trials showed that high-dose vitamin C (2–3 g/day) is recommended to restore normal plasma concentration [9, 10]. Studies which used high doses of vitamin C (3–10 g/day) showed advantageous effects on biological and clinical outcome in critically ill patients [11, 12]. Based on the safety profile and molecular characteristics of vitamin C in critically ill patients and the controversial results of the previous studies, we aimed to evaluate the effect of high dose intravenous administration of vitamin C on the mortality of critically ill patients.

## Methods

After obtaining ethics committee approval and getting informed consent from patients or their legal guardian (when the level of consciousness of patients was low), 80 critically ill patients with severe pneumonia were enrolled in this randomized double-blinded clinical trial. (Trial registration number: IRCT, 20190312043030 N1).

All patients who were admitted to the intensive care unit (ICU) of a university-affiliated hospital in the northwest of Iran with the diagnosis of severe pneumonia were enrolled in this study from May 2019 till Dec 2019. Patients with a CURB-65 score > 3, one major criterion, or ≥ 3 minor criteria were considered as severe pneumonia. Exclusion criteria were age less than 18 and more than 80 years old, renal insufficiency, history of vitamin C usage during past 48 h, allergy to vitamin C, pregnancy or breastfeeding, life expectancy of fewer than 24 h, previously complicated with end-stage lung disease, end-stage malignancy, glucose-6-phosphate dehydrogenase deficiency, diabetic ketoacidosis, active kidney stone disease, and participation in another clinical trial at the same time. The severity of pneumonia was diagnosed based on CURB-65 (confusion, uremia, respiratory rate, BP, and age ≥ 65 years) and PSI index.

We constructed 6 blocks in AABB, BBAA, ABAB, BABA, ABBA, and BAAB using four blocks. We assigned 1 to 6 for each block. Then, using the random number table, based on the sample size, 20 units of 4 blocks were selected so that we considered having 40 people in the control group and 40 people in the intervention group. Therefore, we did block randomization. In this study, patients, clinical caregivers, and data analyzers were not aware of grouping. Patients were randomly assigned to one of the following groups: intervention group in which patients received standard treatment plus 60 mg/kg/day vitamin C as a continuous infusion for 96 h. Patients in the control group received standard treatment plus intravenous infusion of normal saline as the placebo in the same volume. Standard pneumonia therapy included empirical antibiotic therapy before the administration of appropriate antibiotics based on the bacteria isolated on laboratory testing, as well as adjunct respiratory therapy. Patients’ demographic characteristics were recorded during the study period. Sequential organ failure assessment (SOFA) was assessed during the study and acute physiologic and chronic health evaluation (APACHE II) was assessed on the first day of ICU admission. We evaluated patients for any complication during intravenous infusion of vitamin C including hypotension (systolic blood pressure less than 70 mmHg or 30% decrease compared to the baseline), tachycardia (increase more than 20% compared to baseline, or heart rate more than 120/min), nausea/vomiting, and hypernatremia. If any of the aforesaid complications were seen, the rate of administration of vitamin C was decreased by 50% and if it continued, the infusion was stopped. Serum level of vitamin C was noted at baseline and 48 h after vitamin C administration. Duration of mechanical ventilation, ICU length of stay, PaO_2_/FiO_2_, and mortality rate were noted for all patients until the 28th day.

The sample size was calculated based on the mortality rate reported by Marik and colleagues [13], such that a minimum sample size of 28 per group was calculated in order to detect at least a 31.9% difference in proportions of the mortality, P_int_ = 8.5%, P_control_ = 40.4, and to fulfill a minimum statistical power of 80% and type 1 error of 5%. Data were analyzed using SPSS 17 software and reported as mean ± standard deviation for the continuous variables, and percentage for discrete variables. Non-continuous variables were analyzed with Chi-square and continuous variables were analyzed with T-test. Organ dysfunction indices based on SOFA/APACHE scores were compared by regression correlation and T-test. A *P*-value of less than 0.05 was considered significant.

## Results

**Fig. 1:**
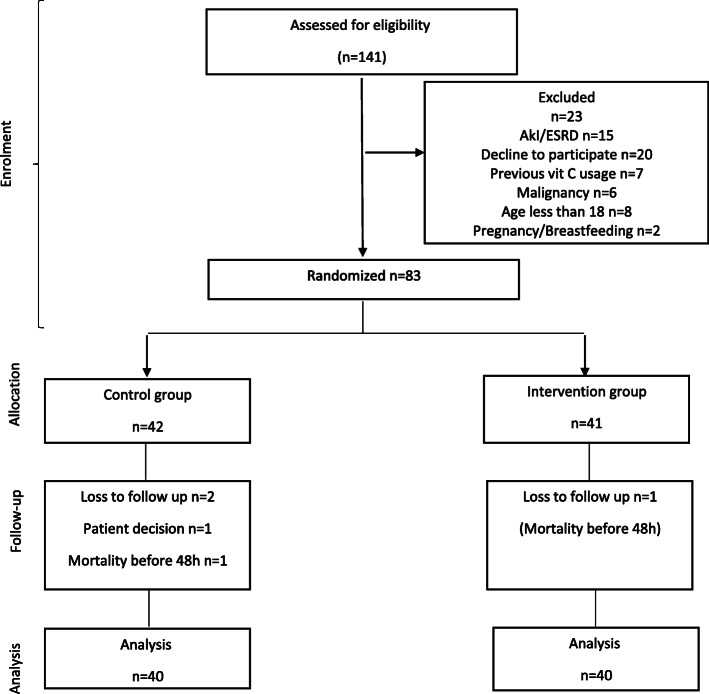
Flow diagram of the study

A total of 141critically ill patients with severe pneumonia were eligible for this study. Fifty-eight patients were excluded from the study and 83 patients were randomized into two groups. Three patients were withdrawn due to mortality before 48 h and patient refusal to participate in the study (Fig. 1). Finally, two groups of 40 patients were analyzed in this randomized trial. The mean age of patients was 57.95 ± 12.9 years with a male/female ratio of 46/34. Frequencies of hospital-acquired pneumonia, community-acquired pneumonia, and ventilator-associated pneumonia were 34, 21, and 25, respectively. The causes of typical bacterial pneumonia in our study were *Streptococcus pneumoniae* (pneumococcus) (21%), mixed (15%), haemophilus influenza (1.6%), *Staphylococcus aureus* (4%), Enteriobacteriaceas (35%), moraxella catarrhalis (0.4%), unknown (23%).

Demographic characteristics of patients during the study are shown in Table 1. Vasopressor was used in 48 patients and the only used vasopressor was norepinephrine. Duration of mechanical ventilation and vasopressor use was significantly less in the intervention group (*p* < 0.001 and = 0.003, respectively). Our results showed that the SOFA score decreased during the study in both groups but the level of this reduction was more in the intervention group than the control group. Moreover, its difference was significant at 72 and 96 h between the two groups (*p =* 0.01 and < 0.001, respectively). The results were the same for procalcitonin, C-reactive protein, and PaO_2_/FiO_2_ levels in two groups after 24 h. Baseline levels of vitamin C in both groups did not have a significant difference but the level increased in the intervention group and decreased in the control group during the study (Table 2). Totally, 17 patients died in this study which 6 of them were in the intervention group. Results showed that mortality was insignificantly lower in the intervention group (*p =* 0.17). Three patients showed hypotension and tachycardia during the administration of vitamin C which was self-limited by decreasing the dose of vitamin C. The frequency of acute kidney injury in the two groups did not have a significant difference (*p =* 0.12).

**Table 1:** Demographic characteristics of patients

Variable		Control	Intervention	***P*** value
**Age** (Years)		58.25 ± 13.1	56.93 ± 12.3	0.47
**Male/Female**		22/16	24/18	0.39
**APACHE**		22.7 ± 4.26	24.5 ± 5.35	0.57
**SOFA**		10.7 ± 2.70	12.54 ± 2.65	< 0.001
**Pneumonia**				0.56
VAP		12	13	
CAP		10	11	
HAP		18	16	
**PSI**		188.52 ± 52 ± 33.87	194.72 ± 29.83	0.38
**CURB-65**		3.55 ± 0.71	3.72 ± 0.67	0.26
**MV duration** (day)		8.92 ± 2.96	4.05 ± 2.29	< 0.001
**ICU LOS (**day)		14.15 ± 3.12	12.77 ± 3.71	0.07
**Vasopressor use** (day)		3.39 ± 1,23	2.28 ± 1.24	0.003
**Vasopressor dose** (microg/min)		8.26 ± 3.58	6.8 ± 3.18	0.14
**Need to mechanical ventilation**^α^		9	5	0.24
**Mortality**		11	6	0.17
**Comorbidities**^a^	**AKI**	22(55%)	16(40%)	*P* = 0.144 X2 = 6.763
	**DM**	11(27.5%)	25(62.5%)	
	**Cardiovascular**	13(32.5%)	18(45%)	
	**Cerebrovascular**	8(20%)	11(27.5%)	
	**Malignancy**	5(12.5%)	3(7.5%)	

*APAHE* acute physiology and chronic health evaluation *SOFA* sequential organ failure assessment *VAP* ventilator associate pneumonia *CAP* community acquired pneumonia *HAP* hospital acquired pneumonia *PSI* pulmonary severity index *CURB-65* (confusion, blood urea > 42,8 mg/dl, respiratory rate > 30/min, blood pressure < 90/60 mmHg, age > 65)

^a^See chart 1

**Table 2:** clinical variables during treatment in two groups

Variable	T0	T24	T48	T72	T96
**SOFA**					
Intervention	12.45 ± 2.65	10.27 ± 2.60	7.1 ± 1.92	3.72 ± 1.75	1.62 ± 1.19
Control	10.7 ± 2.70	8.8 ± 2.65	7.92 ± 2.81	4.85 ± 2.51	3.10 ± 1.61
*P* value	< 0.001	0.01	0.93	0.01	< 0.001
**PCT**					
Intervention	37.25 ± 20.93	26.50 ± 15.30	11.10 ± 6.04	4.02 ± 2.34	1.36 ± 0.79
Control	45.28 ± 28.60	28.07 ± 17.48	16.27 ± 11.71	7.72 ± 7.29	2.82 ± 1.39
*P* value	0.15	0.67	0.01	< 0.001	< 0.001
**CRP**					
Intervention	27.40 ± 11.66	36.60 ± 13.85	14.97 ± 7.31	6.70 ± 3.61	2.30 ± 0.66
Control	24.27 ± 12.83	45.1 ± 19.50	24.32 ± 12.72	11.47 ± 7.51	4.79 ± 1.78
*P* value	0.25	0.02	< 0.001	< 0.001	< 0.001
**Pao2/Fio2**					
Intervention	116.42 ± 27.60	191.49 ± 25.60	224.62 ± 23.40	262.87 ± 30.59	292.65 ± 29.42
Control	160.51 ± 29.70	188.21 ± 26.61	208.50 ± 28.88	229.95 ± 33.10	257.20 ± 33.73
*P* value	0.99	0.6	< 0.001	< 0.001	< 0.001
**Vitamin C**					
Intervention	20.63 ± 12.74				79.20 ± 26.42
Control	22.77 ± 13.56				16.38 ± 10.33
*P* value	0.47				< 0.001

*SOFA* Sequential organ failure assessment

*PCT* Procalcitonin (microgram per liter)

*CRP* C reactive protein (milligram per liter)

VIT C (milligram per liter)

## Discussion

The main finding of this study is that administration of high-dose intravenous vitamin C in critically ill patients with severe pneumonia is safe. This treatment is associated with decreasing in the duration of mechanical ventilation and vasopressor usage and also improvement in oxygenation with a concomitant decrease in pulmonary severity indices without any significant decrease in ICU length of stay and mortality.

Vitamin C is known to be used for treating cancer and respiratory viral infections. However, new information regarding the pharmacokinetic properties of vitamin C and results of recent studies have raised interest in the utilization of high-dose vitamin C in critically ill patients [14]. Previous studies showed that treatment with vitamin C decreased procoagulant and proinflammatory markers in the respiratory system which resulted in lower lung injury [15]. Vitamin C can also diminish the sequestration of neutrophils, improve alveolar fluid clearance, and maintain lung barrier function [16]. Moreover, vitamin C can counter oxidative stress by decreasing hydrogen peroxide, superoxide anion, and nitric oxide levels [17]. So, vitamin C not only increases bacterial killing potency in the early stages but also modulates the immune response in later stages of the disease. This evidence was supported by our results which showed that vitamin C administration significantly diminished the inflammatory and severity markers of critically ill patients [18]. Results of the CITRIS-ALI randomized clinical trial showed that a 96-h infusion of vitamin C compared with placebo in the patients with sepsis did not significantly improve organ dysfunction scores or alter markers of inflammation and vascular injury [12]. Our results showed a significant improvement in the SOFA score on 72 and 96 h which may result from the deteriorated conditions of certain patients, leaving the survived patients with less severe conditions for statistical analysis at the primary endpoints. Now a randomized controlled trial to ascertain the effect of vitamin C on the composite endpoint of death or persistent organ dysfunction at 28 days in patients with sepsis is under investigation which can help to better define of this problem [19].

Results of an interesting study showed that nearly 70% of critically ill patients had hypovitaminosis C despite receiving standard ICU nutritional support which emphasized the importance of its substitution in critically ill patients [2]. Results of a recently performed RCT showed that administration of 15 g/day of intravenous vitamin C for 96 h in 167 patients with ARDS due to sepsis showed a decrease in mortality [12]. This trial used the same duration but much higher doses of vitamin C compared to our trial with larger sample size; this can explain its results regarding mortality. One important point regarding the administration of vitamin C is the fact that it is not possible to restore normal levels with oral supplementation due to saturable absorption kinetics and reduced absorption in critical illness; so, intravenous administration is necessary. The other point is the time to start vitamin C therapy. The earlier the treatment is started, the better are the results. Delayed starting in patients evolves into a phase of irreversible multi-system organ failure and at this time treatment may be futile. Hemila et al. evaluated six trials of vitamin C administration in ICU in a recently published meta-analysis. In three trials in which patients needed mechanical ventilation for over 24 h, vitamin C shortened the duration of mechanical ventilation by 18.2% (95% CI 7.7 to 27%; *p* = 0.001). They recommended that the effect of vitamin C should be investigated in more trials based on low cost and decrement in ICU length of stay [20]. Cai et al. showed that vitamin C can improve the outcome in pneumonia due to influenza virus by its effect on inhibition of CORT synthesis which reduces the susceptibility to influenza viral infection [21]. Regarding critically ill COVID-19 patients with pneumonia with a high mortality rate, it seems that timely administration of high-dose intravenous vitamin C has been particularly effective by inhibiting the production of cytokine storm due to COVID-19 [22].

Results of this study showed that administration of intravenous vitamin C can increase the concentration of vitamin C in critically ill patients after 72 h. We showed that the level of CRP, PCT, SOFA score, and P/F ratio was significantly improved after 72 h. Combining the results, we can conclude that a significant increase in levels of vitamin C can result in significant improvement in pulmonary organ function and decreasing in inflammation/ infection in critically ill patients which is similar to previous results.

The present study has several limitations. Previous studies regarding the effect of vitamin C in critically ill patients are mostly done in septic patients. There are few studies on the effect of vitamin C on pneumonia and because of the low number of trials, we first wanted to show the effect of administration of vitamin C in patients with pneumonia, then evaluate the effect of vitamin C on different subgroups of pneumonia. Thus, the results of this study cannot be generalized to all critically ill patients with pneumonia, different comorbidities, or surgical problems. Also, 15 patients in the control group and 10 patients in the intervention group received corticosteroids for shock reversal, resulting in a diminution in the vasopressor dose which could interfere with our results.

## Conclusion

Our results showed that the intravenous administration of a relatively high dose of vitamin C to critically ill patients with severe pneumonia is safe and can decrease inflammation, mechanical ventilation duration, and vasopressor use without any significant effect on mortality. However, based on the aforesaid limitation, future large randomized controlled studies are needed to evaluate the optimal dose and duration, and probable adverse effects of vitamin C in critically ill patients with severe pneumonia.

## Data Availability

The datasets used and/or analyzed during the current study are available from the corresponding author on reasonable request.

## Abbreviations

CURB-65: Confusion, Uremia, Respiratory Rate, Blood Pressure and Age ≥ 65 Years
PSI: Pneumonia Severity Index
ICU: Intensive Care Unit
SOFA: Sequential Organ Failure Assessment
APACHEII: Acute Physiologic and Chronic Health Evaluation
